# Identification of MicroRNAs and Transcript Targets in *Camelina sativa* by Deep Sequencing and Computational Methods

**DOI:** 10.1371/journal.pone.0121542

**Published:** 2015-03-31

**Authors:** Saroj Poudel, Niranjan Aryal, Chaofu Lu

**Affiliations:** 1 Department of Computer Science, Montana State University, Bozeman, Montana, United States of America; 2 Department of Plant Sciences and Plant Pathology, Montana State University, Bozeman, Montana, United States of America; Institute of Botany, Chinese Academy of Sciences, CHINA

## Abstract

*Camelina sativa* is an annual oilseed crop that is under intensive development for renewable resources of biofuels and industrial oils. MicroRNAs, or miRNAs, are endogenously encoded small RNAs that play key roles in diverse plant biological processes. Here, we conducted deep sequencing on small RNA libraries prepared from camelina leaves, flower buds and two stages of developing seeds corresponding to initial and peak storage products accumulation. Computational analyses identified 207 known miRNAs belonging to 63 families, as well as 5 novel miRNAs. These miRNAs, especially members of the miRNA families, varied greatly in different tissues and developmental stages. The predicted miRNA target genes are involved in a broad range of physiological functions including lipid metabolism. This report is the first step toward elucidating roles of miRNAs in *C*. *sativa* and will provide additional tools to improve this oilseed crop for biofuels and biomaterials.

## Introduction

Camelina [*Camelina sativa* (L.) Crantz], also known as false flax or gold of pleasure, is a temperate climate oilseed crop belonging to the mustard family, Brassicaceae [1]. Camelina is an ancient oilseed with a history of cultivation dating to the Bronze Age in Europe, which oil was used as massage oil, lamp fuel, and cooking oil while the leftover meal was used for animal feed [1,2]. Today camelina is generating renewed interest as a second generation oilseed crop for biofuels and there is some limited interest in it for an omega-3 fatty acid source as well due to its high content of α-linolenic acid [2,3]. Camelina can be found growing in a wide range of climatic and soil conditions including low fertility or saline soils, and is relatively tolerant of cold weather and drought [1,4]. The oil content of camelina seeds can vary considerably between genotypes from 30–45% leading to a wide range of potential oil yields reported [5,6]. Camelina is considered to be a low input crop compared to other oilseeds for water usage, fertilizer and pesticide requirements [1,4]. The short life cycle of camelina at 85–100 days also allows it to fit into rotation with crops like winter wheat without disrupting existing planting times.

In spite of having the potential to be an industrial platform for biofuels and biotechnological materials [3], it remains challenging to improve camelina seed characteristics especially seed yield and oil content. Efficient improvement of camelina may be hampered by its scarce genetic resources such as limited gene maps [5], and the allohexaploidy of the camelina genome [7,8]. However, the close relationship of camelina to the model plant *Arabidopsis thaliana* [9] and the recently developed camelina genome sequence [8] and the seed transcriptome databases [10,11] will aid in gene identification to enhance the ability for camelina improvement.

MicroRNAs (miRNAs) have emerged as one of the important regulators of gene expression in plants and animals [12]. MiRNAs are short 20–24 nucleotide (nt) non-coding RNA sequences. To date, the databases (primarily miRBase) contain 35,828 mature miRNA from 223 species [13]. MiRNAs control gene expression by binding to specific complementary mRNA targets in the untranslated regions (UTR) or the coding sequence (CDS), leading to mRNA degradation or translational inhibition [14]. This regulation plays critical roles in various biological and molecular processes in plants such as development [15] and stress responses [16,17]. The roles of miRNAs in regulating lipid metabolism have also been implicated in a number of studies involving oilseed crops [18–20]. It is also possible to use miRNA-based strategies, e.g., artificial miRNAs to alter gene expression and modify seed oil composition [21].

In the present study, we have identified known and novel miRNAs in *C*. *sativa* through deep sequencing of small RNAs extracted from leaves, flower buds and developing seeds. The targets of these miRNAs were predicted in silico, which have a broad range of biological functions. This study will serve as the starting point to investigate the roles of miRNAs in camelina, and to provide additional tools to improve this important bioenergy crop.

## Materials and Methods

### Plant Growth and Sample Collection

A *Camelina sativa* variety, Suneson, released by Montana State University, was used in this study. Plants were germinated as 5 seeds to a 6" or 8" pot and grown in a 1:1 mix of MSU soil (equal parts by volume of loam soil: washed concrete sand: Canadian sphagnum peat moss with AquaGro 2000 G wetting agent blended in at 1 lb/cubic yard of soil. Aerated steam pasteurized at 70°C for one hour) and Sunshine Mix #1 (Bellevue, WA, USA). Greenhouse conditions were 22°/18°C +/- 1°C for day/night temperatures, a relative humidity of 30%, and a 16 hour photoperiod of natural lighting supplemented when necessary by season.

Young leaves, flower buds and developing seeds at 13 (initial stage of storage accumulation) and 19 (peak stage of storage accumulation) days after flowering (DAF) [22] were collected, and immediately frozen in liquid nitrogen. Samples were stored in -80°C until RNA extraction.

### RNA Extraction and Sequencing

Total RNA from collected samples were extracted using Norgen miRNA extraction Biokit (Thorold, ON, Canada), following manufacturer’s instructions. Total 50 μl RNA were extracted using the kit. About 3–5 μl were used to evaluate the quality by electrophoresis on a 1% agarose gel. One μl was used to examine the RNA quantities using NanoDrop (Nanodrop Technologies, Wilmington, DE, USA). A minimum of 3 duplicates were obtained and quantity was accessed using the average of 3 runs. High quality RNAs were sent for small RNA sequencing to LC Sciences (Houston, TX), where they were analyzed again by a Bio-analyzer. To generate small RNA libraries, following steps were taken: since most mature miRNAs have a 5'-phosphate and a 3'-hydroxyl group, the Illumina adapters in TruSeq Small RNA kit (kit guide) were directly and specifically ligated to miRNAs. The 3’ adapters were ligated followed by 5' adapters. Reverse transcription was performed to obtain the single stranded cDNA. Purified single stranded cDNAs were then PCR amplified using a common primer and a primer containing one of 48 index sequences for cluster generation which was sequenced for 50 bases on Illumina HiSeq 2000 using single end read. Reads were obtained considering only 3' adapters omitting the reading of 5' adapters.

### Primary Analysis of the Deep Sequencing Datasets

S1 Fig. summarizes the overall analysis steps. The raw reads (50 nt) obtained only contained 3' adapters, thus the reads were clipped to remove 3’ adapters. Clipped reads were further trimmed to discard sequences of low quality (phred value <26) and those having less than 15 nt and higher than 27 nt using fastx-toolkit (http://hannonlab.cshl.edu) using default settings (Table 1).

**Table 1:** Raw and filtered read counts from sequencing *C*. *sativa* sRNAs.

Sample	Unfiltered reads	Filtered reads	%
Leaves	9,856,027	7951133	80.7
Flower buds	9,857,386	8321249	84.4
Seed-13	9,148,826	8938448	97.7
Seed-19	10,596,879	9135921	86.2
**Total**	39,459,118	34346751	87.0

### Identification of miRNAs

Filtered reads (15–27 nt) were first collapsed so that only unique reads were obtained removing all the redundancies using in-house C++, python script and fastx-toolkit. These unique raw reads were blasted against the *C*. *sativa* genome using bowtie2 [23] to obtain reads that were only present in the genome. The reads obtained were then mapped to the reference pre-miRNA and miRNA databases: Viridiplantae miRNA database (miRbase: Release 21) [13] and plant miRNA database (PMRD) [24]. Bowtie1 [25] was used to find unique known miRNAs. Perfectly matched miRNAs were considered to be known miRNAs. Bowtie1 [25] with at least ≤3 mismatches was used to find the candidate novel miRNAs. The raw sequences that were not identified earlier were used to BLAST against the complete mature and precursor miRNA reference databases to find the candidate miRNAs. MiRNAs with abundance of higher than 80 of total reads were extracted for further analysis. Both identified known miRNAs and candidate novel miRNAs were mapped to the *C*. *sativa* genome to extract pre-miRNAs using in-house python script. A window size of 200 was used to consider for the pre-miRNA, i.e. 200-nt up and downstream of the matched sequences in the genome were extracted. Candidate pre-miRNAs were further analyzed through its secondary structure using mfold [26] and Vienna package [27] using default settings to find the structurally defined secondary structure of each miRNAs. We defined the secondary structural features as [28] with slight modification: MFE less than -20 kcal/mol, stem-loop size less than 210nt, and all the candidate miRNAs should at least have 75% sequence complementary to their counterparts.

We have deposited our data in the NIH short read archive (SRA). Data can be accessed by these accession numbers: SRS776891, SRS780909, SRS778709, and SRS781487.

### Experimental Verification of miRNA Expression using Reverse-Transcriptase (RT) PCR

Novel miRNAs and selected known miRNAs were experimentally verified for their presence in the transcriptome. Total RNA extracted using Norgen miRNA extraction Biokit (Thorold, ON, Canada) were used to synthesize cDNA using iScript cDNA synthesis kit from Bio-Rad (Berkeley, CA, USA). Primers (S1 Data) were designed to amplify the whole secondary structure of novel miRNAs and the stem loop of known microRNAs [29]. PCR was run to amplify the cDNA using following parameters: denaturation for 2 minutes at 95°C followed by 31 cycles of 30 seconds at 95°C, 30 seconds at 57°C and 1 minute at 95°C. The final elongation or extension was done at 72°C for 5 minutes. The products were then run at 2.5% agarose gel and screened under ultra violet light.

### Prediction of miRNA Targets

Unique known and novel miRNAs were mapped to *C*. *sativa* coding sequences (CDS) to find the target gene using the server at www.camelinadb.ca [8]. Targeted genes that had score > = 25 (equivalent to expect value of 1) were extracted using in-house python script. Functions of each targeted genes were identified using *C*. *sativa* protein database [8] using in-house python script. In addition, Gene Ontology (GO) terms for cellular components, biological processes and molecular functions of miRNA targets were determined using in-house python script. To identify the GO terms, identified genes were BLASTed against the Arabidopsis Gene Ontology database (www.arabidopsis.org, 2014) and the gene ontology consortium (geneontology.org). In addition, target searches were performed against the Arabidopsis acyl-lipid pathway database (ARALIP: http://aralip.plantbiology.msu.edu/pathways/pathways) [30], www.camelinadb.ca [8] and camelina lipid gene database (http://camelina.cgb.indiana.edu/lipidgenedb.html) to identify miRNA targets that are involved in lipid metabolism.

## Results and Discussion

### Sequencing and Characterization of Small RNA Transcriptomes in Camelina

To identify the known and potential novel miRNAs in *C*. *sativa*, small RNA libraries prepared from young leaves, flower buds and developing seeds at 13 and 19 DAF (seed-13, seed-19) were sequenced by Illumina technology, resulting in a total of 39,459,118 reads (Table 1). After removing the adapters and filtering out low quality sequences, a total of 34,346,751 reads (87%) of 15–27 nt in length were obtained, from which 6,309,099 (18.37%) were unique reads (S1 Table). Other types of RNAs like tRNA, rRNA, snRNA were not filtered and identified as the purpose of this project was to identify miRNAs. However, while identifying novel miRNAs, other non-coding small RNAs such as those mentioned above were filtered using various available market tools, e.g., tRNAscan-SE server [31] and rfam server [32].

**Fig 1:**
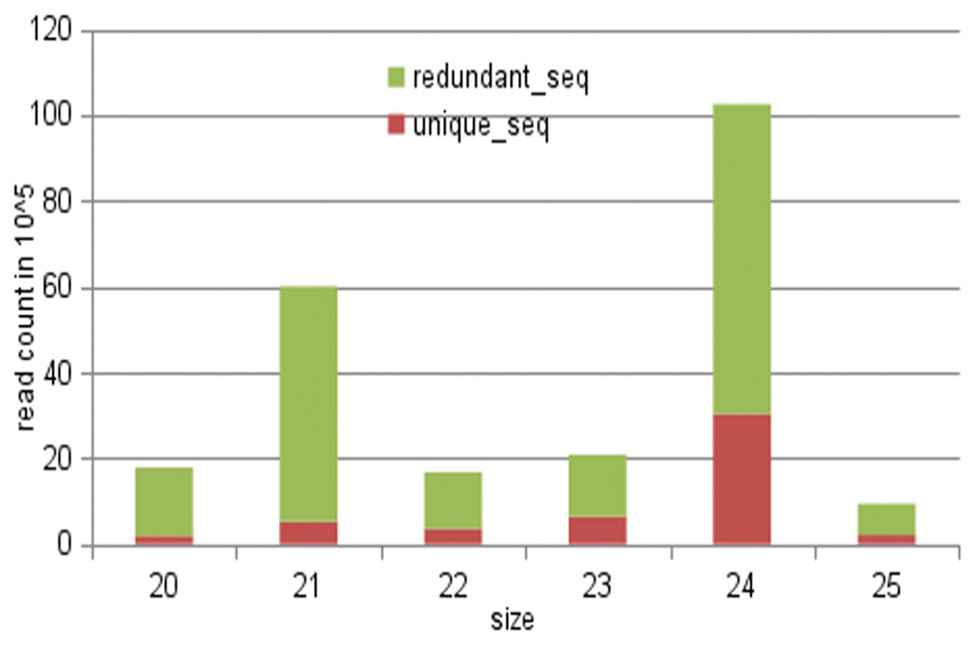
Total number of redundant and unique reads in the sRNA libraries of *Camelina sativa*.

Fig. 1 summarizes the total unique and redundant sequences in the libraries with different sizes. The length distribution of these sRNA reads indicated that the majority of sequences are within 21 and 24 nt, a typical size range for Dicer-like (DCL)-derived miRNA products [33]. Among which, the abundance of 21 and 24-nt long miRNAs were significantly higher than others. The 21-nt small RNAs were most abundance in leaves and buds libraries, accounting for 10.5% and 13.7% respectively of the sequence number, whereas the 24-nt sequences were the highest in seed-13 and seed-19 libraries, accounting for 33.8% and 29.7%, respectively (S1 Table). Subsequently, redundant sequences were removed or collapsed to obtain unique reads, which accounted for 18.37% of the total reads. Among which, the 24-nt sequences were the most abundant in all samples: 45.8% for buds, 28.5% for leaves, 46.2% for seed-13 and 55.8% for seed-19. In summary, unique sequences between 20 to 25-nt ranged from 4% to 56% of the total sequence numbers in each libraries (S1 Table).

We then aligned the unique sequences against the *C*. *sativa* genome [8]. This analysis showed that nearly 80% (5,044,275) of the unique sRNA sequences could be mapped to the camelina genome. Of these 5 million reads, 439,624 (67.65%) were identified in leaf, 877,835 (79.14%) in bud, 1,613,190 (79.64%) in seed-13 and 2,113,626 (83.73%) in seed-19 libraries.

### Identification of Conserved miRNAs in Camelina

A number of studies have reported that mature miRNAs are conserved across different plant species [34,35]. To identify miRNA homologs in camelina, unique raw reads that were mapped to the camelina genome: 877835, 439624, 1,613,190 and 2,113,626 from four libraries (buds, leaves, seed-13, and seed-19) were used to search the mature Viridiplantae unique miRNAs obtained from miRBase 21 (http://www.mirbase.org/; Released June, 2014) and mature plant miRNAs obtained from PMRD: plant microRNA database (http://bioinformatics.cau.edu.cn/PMRD/; June, 2014) [24], which contained 8,496 and 10,898 mature miRNAs, respectively. A total of 15,372 unique mature miRNAs were extracted from both databases for homology searches. To identify conserved miRNAs, only sequences that exactly matched in size and nucleotide composition were considered. The search resulted in a total of 3,497,756 reads as known miRNAs which accounts for 69.34% of total reads that were mapped to the camelina genome. Of which, 338,145 (76%) were identified in leaf, 852,459 (97%) in bud, 886,156 (55%) in seed-13 and 1,420,996 (68%) in seed-19 libraries, respectively. Initially, we identified 737 known mature miRNA hits that belonged to 108 families. These hits were used to find the secondary structure (pre-miRNA) of each identified miRNAs. We identified 97 unique precursor miRNAs (i.e., miRNA/miRNA* duplex), so a total of 194 mature miRNA/miRNA* were discovered through the secondary structure analyses. Another 13 conserved miRNAs were also identified although they did not meet the minimum criteria for forming secondary structure. In summary, a total of 207 known miRNAs belonging to 63 families have been identified in camelina (S2 Data). S2 Table summarizes all the known miRNAs identified along with their sequences and the plant species in which they have been reported to be present.

The member numbers in different families differed greatly. MiR156 and miR169 represent the largest families with 11 and 7 members, respectively. Of the remaining, 3 families have more than 3 members, 10 families have 2 to 3 members, and 45 members are represented by only a single member. The expression levels indicated by read abundance also showed vast differences among different families. Among which, miR166 and miR165 were the most abundant conserved miRNA families present in all camelina libraries, accounting for 62% and 21% of the reads in buds, 70% and 12% in leaves, 72% and 21% in seed-13, and 78% and 15% in seed-19 of the total miRNA families (S2 Data). Some other miRNA families that are conserved in a large number of plant species (S2 Table), e.g., miR159, miR158, miR167, miR168, miR156, miR157, miR319, miR396, miR173, miR825, miR2910, miR6300 and miR403, are also highly expressed with over 1,000 reads in each library. This suggests that these miRNAs share common roles of regulating different aspects in plants. Furthermore, results in S2 Data indicate that different members of the same miRNA family are expressed at different levels. For example, in the miR166 family, 6 members were detected in leaves with 0 to 527,386 reads, and in seed-19 with 0 to 1,110,054 reads. Among the members, miR166b has been observed to be the most abundant, accounting for 70%, 62%, 71%, and 78% in leaves, buds, seed-13 and seed-19 libraries, respectively. Similarly, miR165a is the second most abundant miRNA among the members, accounting for 12%, 21%, 21%, and 15% of the total members in leaves, buds, seed-13 and seed-19, respectively. These results suggest that although the same miRNA may regulate certain common developmental or metabolic processes, they may fine tune their control by regulating different expression of specific family members.

### Identification of Novel miRNAs and Pre-miRNAs

The above homology searches identified 3,497,756 camelina sequence reads that perfectly matched known miRNAs in plants. To identify potential novel miRNAs in camelina, the remaining small RNA sequences with characteristic hairpin-like structures were searched against the same reference genome/transcriptome sequences allowing maximum of 3 mismatches. A total of 125 mature reads were identified with abundance of higher than 80, which were used to BLAST against *C*. *sativa* genome to extract miRNA precursors. This resulted in a total of 340 precursors that were subsequently analyzed for their secondary structures using mfold and RNAfold. A total of 5 putative novel miRNAs in 54,414 reads (S3 Table) were identified and subsequently confirmed by BLASTing against the miRBase and NCBI databases to verify that they had no homologs in the databases. Of which, 19,990 were observed in bud, 2326 in leaf, 6548 and 6665 in seed-13 and seed-19 libraries, respectively. Furthermore, identified novel precursors were BLASTed against RNA database through Rfam and tRNAscan-SE server to confirm that they are not homologous to any tRNAs or other non-coding RNAs. Novel miRNAs each had read counts of at least 81 out of total reads at least in one of the libraries. The most abundant novel miRNAs (Csa-miR002) yielded 35,529 reads in total reads count, including 33,350 Csa-miR002 and 2,179 Csa-miR002*. Three of the most abundant novel miRNAs (Csa-miR001, 002, 003) had 3p-arm expressed more than its complementary counterparts. All the novel miRNA are 19 to 21-nt long, as was observed for conserved miRNAs. All the pre-miRNA sizes are in the range of 52–213 nts with an average length of 121 nts and have the MFE ≤ -20. S3 Table summarizes all the novel miRNAs identified along with their abundances and positions in the genome. Secondary structures of novel miRNAs are presented in S3 Data.

**Fig 2:**
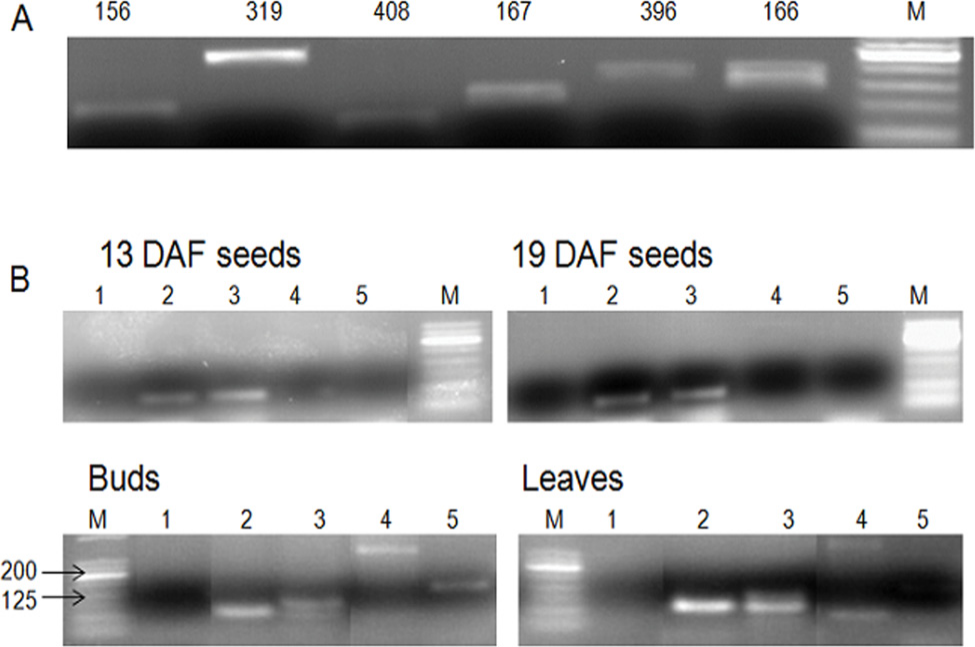
RT-PCR to validate the presence of miRNAs in camelina tissues. (A) Known miRNAs (156, 319, 408, 167, 396 and 166) in leaves. (B) Five novel miRNAs in four tissue samples of camelina. M: DNA ladder.

We also validated the presence of the novel miRNAs along with selected known miRNAs by RT-PCR in different tissues of camelina. Six known miRNAs that are highly expressed among different plant species were selected, including miR156, miR166, miR167, miR319, miR396 and miR408, and all were detected for their expression by RT-PCR of their pre-miRNAs in the leaves of camelina (Fig. 2A). Using similar approaches, we detected the expression of the five novel miRNAs. Four pre-miRNAs could be detected by RT-PCR: pre-Csa-miR002 and pre-Csa-miR003 were detected in all four tissues; pre-Csa-mi004 was detected in leaves and buds, but pre-miR005 was only amplified from bud samples (Fig. 2B). Interestingly, pre-Csa-miR004 has an unusually long sequence of 213nt. Further computational analysis suggested that it could produce a known mature miRNA (miR159a). Pre-miRNA for Csa-miR001 could not be amplified from any tissues though it had high read counts by sequencing. Its expression in camelina needs to be verified by other experimental approaches in the future.

Camelina is an emerging bioenergy and biotechnology crop [3]. There has been no miRNAs sequences reported. In this context, deep sequencing provides a powerful technology to identify miRNAs in camelina. Camelina belongs to the Brassicacae family and is closely related to Arabidopsis [8]. Currently there are 427 and 384 of miRNAs in the miRBase for *A*. *thaliana* and *A*. *lyrata*, respectively. *C*. *sativa* has roughly tripled numbers of genes compared to *A*. *thaliana* [8]. We report here that 207 known miRNAs and 5 novel miRNAs have been identified in *C*. *sativa*. This is lower than expected miRNAs present in camelina given its close relation to Arabidopsis and the strong conservation of miRNAs in plants [35], partly because we have only investigated young leaves, flower buds and two stages of developing seeds of camelina plants grown under normal conditions. Nevertheless, our report represents the first step towards elucidating roles of miRNA in camelina.

### Differential Expression of miRNA in Different Tissues of Camelina

MicroRNAs regulate many biological processes in plants; therefore miRNA expression is highly coordinated and their accumulation patterns may provide clues about their physiological significance [36,37]. Large numbers of sequences obtained by deep sequencing enable the use of read counts in libraries as a reliable source for estimating the abundance of miRNAs [38]. We compared camelina miRNA accumulation levels in leaves, flower buds, developing seeds at 13 and 19 DAF by normalizing read counts of each identified miRNA to the total number of miRNA read counts in each library. The relative abundance showed great variation for some miRNA families. There are 24 miRNAs that were detected with only 1–12 reads in one or more libraries, and miR394 was detected in seed-13 only at 171 reads (S2 Data). Table 2 lists 24 miRNA families with over 50 read counts that showed at least 2-fold changes in reproductive tissues (flower buds and developing seeds, seed-13 and seed-19) compared with their respective levels. For example, miR166, miR165, miR319, miR408, miR827 and miR858 all have increased abundance in flower buds and developing seeds than in leaves, while were predominantly detected in leaves. Some miRNAs, such as miR403, miR396, miR4995 and miR6173 had decreased levels in buds or developing seeds compared with their levels in leaves. We noticed that accumulation levels of different members in a given miRNA family also varied. For example, the total read counts of miR159 in leaves and buds were insignificantly different at 18,462 and 21,199, respectively, however, read counts of Csa-miR159c in buds was 3,534 compared to only 52 in leaves. Although these results need to be experimentally validated, differential expression of miRNAs suggest their different roles in different developmental stages. The clearly different expression levels of different members in a given miRNA family may imply their functional divergence.

**Table 2:** Camelina miRNA with read counts changed significantly in different tissues.

miRNA family	Reads count				Fold change*			
	Leaves	Buds	Seed-13	Seed-19	Buds/Leaves	Seed-13/Leaves	Seed-19/Leaves	Seed-19/13
miR156	1627	777	5536	10364	-2.1	3.4	6.4	
miR159	18462	21199	16976	34027				2.0
miR160	277	530	32	179		-8.7		5.6
miR164	107	89	150	50			-2.1	-3.0
miR165	40625	179353	185703	214186	4.4	4.6	5.3	
miR166	236851	528018	634519	1110941	2.2	2.7	4.7	
miR167	3253	1726	13362	6405		4.1	2.0	-2.1
miR168	656	1407	2373	1180	2.1	3.6		-2.0
miR169	269	183	213	109			-2.5	-2.0
miR170	46	61	73	93			2.0	
miR171	388	453	193	355		-2.0		
miR172	817	948	594	223			-3.7	-2.7
miR173	422	557	868	1031		2.1	2.4	
miR319	69	3988	378	2514	57.8	5.5	36.4	6.7
miR393	74	38	68	30			-2.5	-2.3
miR395	74	63	344	32		4.6	-2.3	-10.8
miR396	2995	1486	710	923	-2.0	-4.2	-3.2	
miR398	12	11	796	479		66.3	39.9	
miR403	5775	4245	2018	3251		-2.9		
miR408	4	8	173	23	2.0	43.3	5.8	-7.5
miR825	5261	4227	1198	2593		-4.4	-2.0	2.2
miR824	82	92	157	698			8.5	4.4
miR827	7	196	239	647	28.0	34.1	92.4	2.7
miR858	13	74	211	500	5.7	16.2	38.5	2.4
miR2910	2667	1824	1408	835			-3.2	
miR4995	228	95	9	7	-2.4	-25.3	-32.6	
miR6300	4981	89137	7872	12800	17.9		2.6	
miR6173	75	7	1	1	-10.7	-75.0	-75.0	

* Only fold changes > = 2 are shown. Negative numbers indicate decreased change over 2-fold.

We also examined the miRNA differential expression during seed development by comparing abundances in seed-13 and seed-19 libraries, which were prepared from small RNAs at 13 and 19 DAF, representing the initial and peak stages of storage synthesis, respectively [22]. Apart from miR394 mentioned above that was only present in seed-13 with over 171 read counts, some miRNAs with less read counts showed dramatic difference between the two seed developmental stages (seed-13/seed-19) include: miR399 (22/8), miR828 (7/35), miR857 (0/12), miRf10010 (13/36) and miR161 (3/12). For miRNAs with over 50 counts, 8 miRNA families (miR159, miR160, miR319, miR824, miR825, miR827, and miR858) increased >2-fold, 7 miRNA families (miR164, miR167, miR168, miR172, miR393, miR395, and miR408) decreased >2-fold in seed-19, compared to seed-13 (Table 2). Previously, expression changes of some miRNAs were investigated in different stages of developing siliques of canola (*Brassica napus*) [39]. Similar to that report, we observed that miR156 also increased (1.9 fold) in the later stage (seed-19) of seed development, and miR390 was not significantly changed either; however, miR167 decreased more than 2-fold in seed-19 compared with that in seed-13, contradicting with a sharp increase at a later stage of siliques in *B*. *napus* [39]. This comparison suggests that although camelina and canola are both oilseed crops in the same family, miRNAs may play different roles during seed development. These results clearly demonstrate that miRNA accumulation changes in the course of camelina seed development, and warrant further research on the roles of miRNAs during seed maturation and storage accumulation by comparing their accumulation at more time points and deeper studies of individual miRNAs.

### Prediction of *C*. *sativa* miRNA Targets

**Fig 3:**
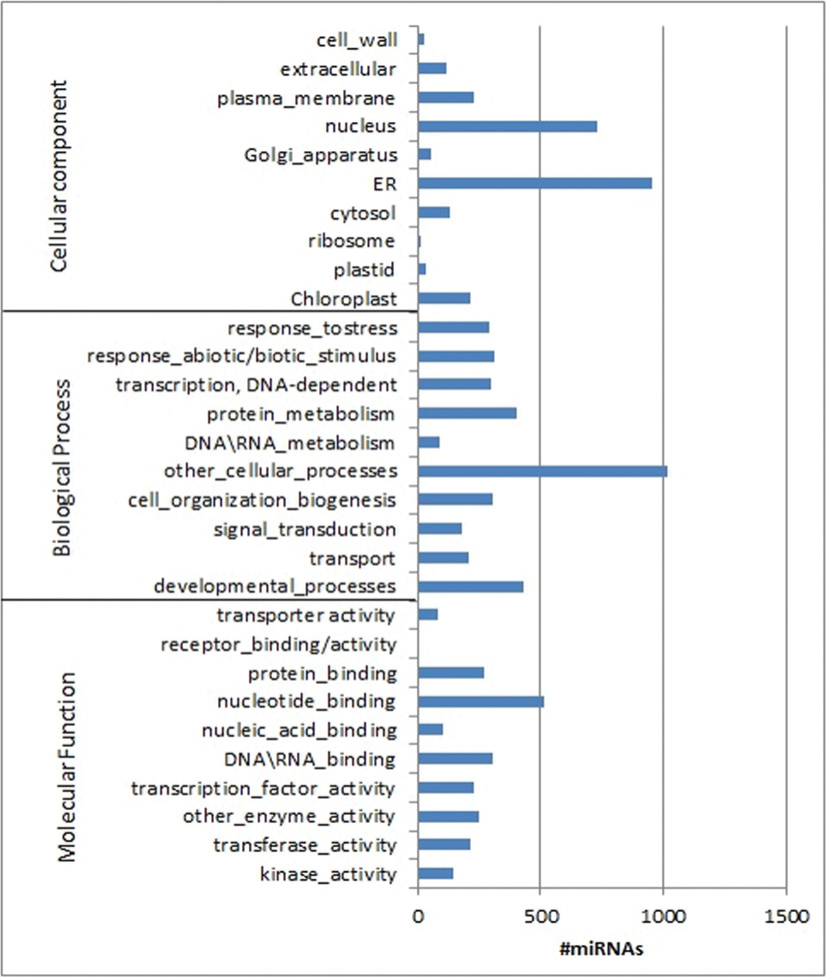
Targets of all identified miRNAs in *C*. *sativa*.

Since most known plant miRNAs regulate gene expression by binding to the protein-coding region of their mRNA targets with nearly perfect sequence complementarity, and degrade the target mRNA in a way similar to RNA interference (RNAi) [40,41], it is critical to predict targets of miRNAs in camelina to gain insights into their regulatory roles. Recently available camelina transcriptomic sequences [8,10] allowed us to predict targets of miRNAs identified in this study. The miRNA sequences were BLASTed against the camelina transcriptome (http://www.camelinadb.ca/), and the target genes were subject to BLASTX against protein databases, followed by Gene Ontology analysis to predict their functions. A total of 4,378 target genes of known miRNAs in camelina, along with their 1,794 Arabidopsis orthologs, are listed in S4 Table. These numbers of target genes are in agreement with the finding that the *C*. *sativa* genome is a triplication of that of *A*. *thaliana* [8]. As reported in previous studies of Arabidopsis microRNA targets [41,42], GO analysis indicated that the target genes in camelina have various functions in protein binding, DNA binding, developmental process, signaling, and as transcription factors (Fig. 3). Similarly, novel miRNAs identified in this study targeted 28 *C*. *sativa* genes that function in development or disease resistance (S3 Table).

Camelina has emerged as a potential bioenergy crop for its seed oils. We are particularly interested in miRNAs that may be involved in regulating lipid metabolism in seeds. To maximize the possibility of identifying potential targets, the parameter of score was decreased to 20, i.e. target genes that had score of > = 20 were considered (see Materials and Methods). Searching for putative target genes of 30 miRNAs with more than 10 reads in developing seed libraries identified 133 candidates that are related to lipid metabolic pathways (S5 Table). The most abundant miRNA (Csa-miR166a) targets genes encoding an acyl-CoA oxidase, which is involved in the first step of fatty acid β-oxidation. This suggests that miRNAs may play important roles in developing seeds to decrease the rate of lipid breakdown. Concurring with this, miRNAs targeting lipases (Csa-miR2910, miR824) were also relatively abundant especially in seed-19, when oil synthesis is actively taking place. Other miRNAs of high read counts were predicted to target genes involved in sphingolipid synthesis (Csa-miR168a), and cuticular wax synthesis (Csa-miR395). Interestingly, genes encoding the 1-acylglycerol-3-phosphocholine acyltransferase (LPCAT) were predicted targets of Csa-miR319a,b in developing seeds, which had significantly increased abundances in seed-19 compared with seed-13. LPCAT is involved in acyl-editing on phosphatidylcholine and is one of the most important factors that determine the composition of fatty acids, e.g., polyunsaturated fatty acids, in seed triacylglycerols [43]. Csa-miR408a, on the other hand, was predicted to target another important gene (*FAE1*) that determines the content of very-long-chain fatty acids in seed oils [44]. The prediction of putative miRNA targets that are involved in fatty acid modification provides valuable insights on the roles of microRNAs in regulating plant lipid metabolism.

## Conclusion

The oilseed crop camelina has been under intensive development for biofuels and industrial oils in recent years. The present study identified microRNAs, including 207 known belonging to 63 families, as well as 5 novel ones that are involved in a wide range of biological and physiological functions. Several miRNAs are computationally predicted to target genes involved in lipid metabolism in seeds. This work is a starting point to elucidate the complex miRNA-mediated regulatory systems in camelina, and to provide additional molecular tools for agronomical improvement. Further studies are necessary to validate the expression and the targets of the miRNAs identified in this report. It is also needed to search for more novel miRNAs that may contribute to the unique attributes of camelina by profiling small RNAs at more time points of seed development and under specific environmental stress conditions.

## Supporting Information

S1 Data PCR primers used for RT-PCR detection of pre-miRNAs. (XLSX)

S2 Data Known miRNAs identified in all samples (leaves, buds and seeds) along with their abundances. (XLS)

S3 Data Secondary structure of novel miRNAs. (DOC)

S1 Fig Flow chart of the methodology applied to identify miRNAs in *C*. *sativa*. (TIF)

S1 Table Size distribution of sRNA reads. (XLSX)

S2 Table Known *C*. *sativa* miRNA and their homolog species reported in miRBase and PMRD. (XLS)

S3 Table Novel miRNAs and their targets in the *C*. *sativa* genome. (XLSX)

S4 Table Known miRNA targets and their functions. (XLS)

S5 Table Predicted miRNA targets related to lipid metabolism in camelina seed. (XLS)
