# Supplementary material for: Identification of MicroRNAs and Transcript Targets in Camelina sativa by Deep Sequencing and Computational Methods
Source: PLoS One. 2015 Mar 31;10(3):e0121542. doi: 10.1371/journal.pone.0121542 (PMC4380411; doi:10.1371/journal.pone.0121542)
Supplement: S3 Data — (DOC) [file pone.0121542.s003.doc]

**Data S2. Secondary structures of novel miRNAs in *C. sativa***

Mature miRNA are colored with red being the novel miRNA and green being its complementary-- secondary structure generated by mfold.

>Csa-pmiR001

Folding bases 1 to 65 of Csa-pmiR001

Initial dG = -37.50

10 20 30

5' TT| C A

CTTTGTCTACA TTTTGGAAAGAGTGATG C

3' GAAACAGATGT AAAGCCTTTTTCGCTAC G

AC^ - C

60 50 40

>Csa-pmiR002

Folding bases 1 to 52 of Csa-pmiR002

Initial dG = -33.40

10 20

-- C C--| T

5' GGGATGGGTCG CCGGT CGCC T

3' CCCTGCTCGGC GGCCA GTGG T

TT T CGT^ C

50 40 30

>Csa-pmiR003

Folding bases 1 to 72 of Csa-pmiR003

Initial dG = -39.80

10 20 30

GG- -| GAAAAA A G

5' TAGGA GCG CGGCGGTCGCT CCT GGGC C

3' GTTCT CGT GCTGCCGGCGA GGG CCCG G

AGA G^ GGC--- - A

70 60 50 40

>Csa-pmiR004

Folding bases 1 to 213 of Csa-pmiR004

Initial dG = -91.00

5'

10 20 30 40 50 60 70 80 90 100

- - A A G GA T TT ------- A - G A C

GGA AG AG AGA GAAGAGCTCCTT AGTTCAA GGAGGGT AGC AGGGT AAGT AAAGCT CT AG

CCT TC TC TCT CTTCTCGAGGGA TTAGGTT CTTCTTA TCG TCCCA TTCA TTTCGA GA TT

C C - - A AG T CT GTAATTT - C G C T

210 200 190 180 170 160 150 140

TC ----| AAT AA

TATGGA CCATAA GCCTTATCA TCAA \

ATACCT GGTATT TGGAATAGT AGTT T

TA TTTT^ --- AC

130 120 110

3'

>Csa-miR005

Folding bases 1 to 86 of Csa-miR005

Initial dG = -34.40

10 20 30 40

G A C UC ----| AAAU

5' AAAGCU CU AG UAUGGA CCAUAA GCCUUAUCA C

3' UUUCGA GA UC AUACCU GGUAUU UGGAAUAGU A

G C U UA UUUU^ AGUU

80 70 60 50
