# Supplementary figures and images for: Identification of MicroRNAs and Transcript Targets in Camelina sativa by Deep Sequencing and Computational Methods
Source: PLoS One. 2015 Mar 31;10(3):e0121542. doi: 10.1371/journal.pone.0121542 (PMC4380411; doi:10.1371/journal.pone.0121542)

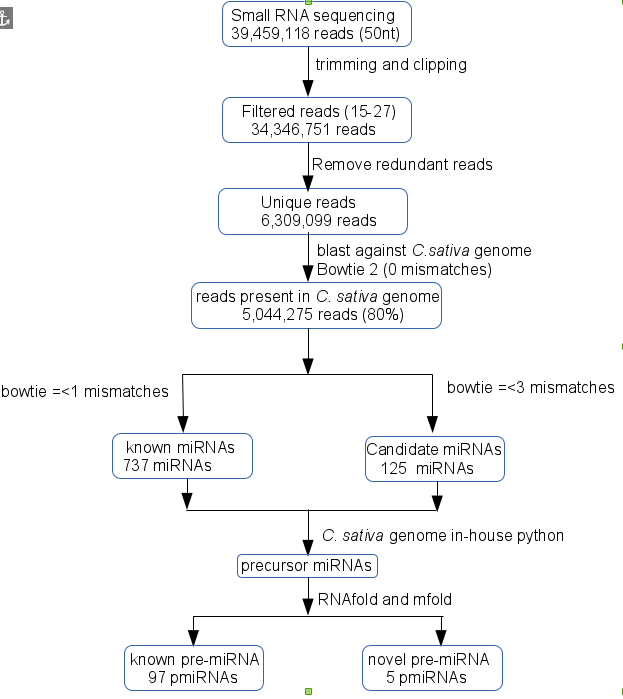

Supplement: S1 Fig — (TIF) [file pone.0121542.s004.tif]
